# Influences of digital media use on children and adolescents with ADHD during COVID-19 pandemic

**DOI:** 10.1186/s12992-021-00699-z

**Published:** 2021-04-19

**Authors:** Lan Shuai, Shan He, Hong Zheng, Zhouye Wang, Meihui Qiu, Weiping Xia, Xuan Cao, Lu Lu, Jinsong Zhang

**Affiliations:** 1 Department of Medical Psychology, Xinhua Hospital Affiliated to Shanghai Jiao Tong University School of Medicine, Xinhua Hospital, 1665 Kongjiang Road, Shanghai, 200092 China; 2 Ministry of Education-Shanghai Key Laboratory of Children’s Environmental Health, Shanghai, China; 3 Shanghai Changning District Mental Health Center, Shanghai, China

**Keywords:** ADHD, COVID-19, Digital media, Mental health

## Abstract

**Objective:**

To explore the influences of digital media use on the core symptoms, emotional state, life events, learning motivation, executive function (EF) and family environment of children and adolescents diagnosed with attention deficit hyperactivity disorder (ADHD) during the novel coronavirus disease 2019 (COVID-19) pandemic.

**Method:**

A total of 192 participants aged 8–16 years who met the diagnostic criteria for ADHD were included in the study. Children scoring higher than predetermined cut-off point in self-rating questionnaires for problematic mobile phone use (SQPMPU) or Young’s internet addiction test (IAT), were defined as ADHD with problematic digital media use (PDMU), otherwise were defined as ADHD without PDMU. The differences between the two groups in ADHD symptoms, EF, anxiety and depression, stress from life events, learning motivation and family environment were compared respectively.

**Results:**

When compared with ADHD group without PDMU, the group with PDMU showed significant worse symptoms of inattention, oppositional defiant, behavior and emotional problems by Swanson, Nolan, and Pelham Rating Scale (SNAP), more self-reported anxiety by screening child anxiety-related emotional disorders (SCARED) and depression by depression self-rating scale for children (DSRSC), more severe EF deficits by behavior rating scale of executive function (BRIEF), more stress from life events by adolescent self-rating life events checklist (ASLEC), lower learning motivation by students learning motivation scale (SLMS), and more impairment on cohesion by Chinese version of family environment scale (FES-CV). The ADHD with PDMU group spent significantly more time on both video game and social media with significantly less time spend on physical exercise as compared to the ADHD without PDMU group.

**Conclusion:**

The ADHD children with PDMU suffered from more severe core symptoms, negative emotions, EF deficits, damage on family environment, pressure from life events, and a lower motivation to learn. Supervision of digital media usage, especially video game and social media, along with increased physical exercise, is essential to the management of core symptoms and associated problems encountered with ADHD.

## Introduction

China was hit by an outbreak of the novel coronavirus disease 2019 (COVID-19) at the end of 2019, and the government implemented a policy of home quarantine to control the infection rate by the end of January 2020, which required citizens to stay at home [17]. The call for quarantine caused schools to shut down and resulted in the abruptly transition to online educational class for continuation of the upcoming academic year [17]. Numerous studies suggested that online classes were considered during the quarantine period, with emphases placed on learning, structure routine, well-being, and social bonds [5]. However, online learning might be particularly difficult for students with pre-existing mental health conditions, such as attention deficit hyperactivity disorder (ADHD) [14].

ADHD is one of the most common mental and behavior disorders among children and adolescents with recent estimates from a meta-analysis indicating that the prevalence in China was 6.26% [42]. Both home quarantine and online classes brought new challenges to families with ADHD children. Some patients experienced interruption of treatment due to the inconvenience of seeking medical assistance while the quarantine policy was in effect. A previous study showed that children with ADHD encountered both increased symptoms and negative emotions during the COVID-19 outbreak [49]. Online classes required the internet and a digital device, which led to free-use of those digital devices and the internet. As a result, many parents were unable to supervise children due to their work responsibility. Current research suggests that one of the core characteristics of ADHD is executive function (EF) impairments, especially inhibition [3, 34]. Those with ADHD, who showed lack of self-control, had a higher rate of problematic digital media use (PDMU) than control children [18]. The combination of home quarantine, online class, and lacking of supervision became a hotbed for PDMU during the COVID-19 pandemic.

Studies have suggested that there is a close relationship between PDMU and attention deficit [43], behavior problems [2] and emotional problems [13]. Some reviews also mentioned PDMU had negative impacts on academic performances, social interactions and family relationships [22, 30]. Digital media products included television, computer, cellphone, video game and internet, which were all considered to be screen-based behaviors. The problematic use behaviors included extended use time, after-dark use, and pornography viewing, which affects living and learning function [22, 30]. However, few studies have simultaneously explored the relationship between PDMU and ADHD symptoms, negative emotions, EF, learning motivation, and family environment.

We hypothesize that during the epidemic quarantine, ADHD children with PDMU would have more attention, behavioral and emotional problems, along with more EF impairments, more stress, less learning motivation, and more adverse effects on their family environment, as compared with the ADHD children without PDMU.

## Method

### Participants and procedures

Participants in this study were ADHD patients aged 8–16 years who had visited a medical psychological outpatient clinic at a general hospital in Shanghai, China, between April and May of 2020, when the COVID-19 epidemic broke out. All participants met the diagnostic requirements for ADHD based on the DSM-V criteria using semi-structured interview tool as clinical diagnostic interviewing scale (CDIS) [4, 45]. Children with major sensory-motor disorders, history of brain damage, epilepsy, diagnosis of autism spectrum disorder were excluded. The information of this study was presented to 208 patients and their parents with a final 192 (92.3%) patients who were willing to participate in the survey.

The 192 participants were divided into two groups using the self-rating questionnaire for problematic mobile phone use (SQPMPU) and Young’s internet addiction test (IAT). The ADHD children with reported problematic use behavior with either the mobile phone or internet, scoring higher than the cut-off point in either SQPMPU or IAT, were defined as PDMU, and others were defined as without PDMU. The demographic data of all the subjects were collected including age, gender, living area per capita, with medication treatment or not, with comorbidities or not.

### Measures

*Self-rating Questionnaire for Problematic Mobile Phone Use (SQPMPU)* is used to estimate the dependence symptoms of mobile phone use for Chinese adolescents [40]. The questionnaire includes 16 items, ranking from 1 to 5, which cover three dimensions and includes withdrawal symptoms, effect of physical and mental health, and craving. Following Zheng et al. [50] we used 27 as the cut-off point for problematic mobile phone use [50].

*Young’s Internet Addiction Test (IAT)* consists of 20 questions, measured on 5-point Likert scale (0 = not at all to 5 = always). A higher total raw score indicates higher addiction symptoms [47]. The Chinese version of IAT has good reliability and validity [23], and a score of 40 as the cut-off point represents problematic internet use [33].

*Swanson, Nolan, and Pelham Rating Scale (SNAP)*: Parents reported children’s ADHD, oppositional defiant disorder (ODD), conduct disorder (CD) and emotional problem symptoms on a 4-point Likert scale (0 = not at all, 1 = just a little, 2 = quite a bit, 3 = very much). The reliability and validity of the Chinese version of SNAP-IV form are satisfactory among children and adolescents in China [51]. The raw scores of symptoms were used to reflect a scale of severity with higher indicating worse symptoms.

*Behavior Rating Inventory of Executive Function (BRIEF)* is a rating scale for parents to assess behaviors in daily life reflecting the EF in children aged 6–18 years [11]. The instrument consists of 88 items on a 3-point ordered scale that measure eight clinical dimensions of EF: inhibition, shift, emotional control, initiation, working memory, plan, organize and monitor. Those scales form two indexes: behavior regulation and metacognition. A composite score, called global executive composite (GEC), is used to indicate general executive performance. The higher the score, the higher the difficulty of the construct. The BRIEF has been shown to have high concurrent and discriminatory validity and adequate reliability in Chinese children [31].

*Adolescent Self-rating Life Events Checklist (ASLEC)* is widely used in the study of evaluating the occurrence and impact of life events for Chinese youth [44]. The impacts of life event are ranked from 5 levels. The factors in this scale include relationship pressure, learning pressure, being punished, loss and adaption problem. A higher raw score represents a higher degree of the pressure and negative impact.

*The Chinese version of Family Environment Scale (FES-CV)* has 90 items, which evaluate 10 aspects of the family social and environmental characteristics respectively, including cohesion, expression, conflict, independent, achievement, intellectual-cultural orientation, active-recreational orientation, morality, organization and control. The score could be used in the adolescent population to reflect the interaction between each member of the family and the overall characteristics of the family environment [39]. The higher the raw score, the better the family environment, except for the conflict which is a low score for “good”.

*Students learning motivation scale (SLMS)* consists of 20 “yes” or “No” questions, which evaluate four dimensions of student’s distress in learning including initiative, awareness, interesting and goal [9]. A higher raw score indicates more distress for the learning motivation.

*Depression self-rating scale for children (DSRSC)* is used to assess the level of depression in children over 8 years old which consists 18 items rated at 3 levels. The higher the raw score, the more obvious the depression symptoms are. The average score with Chinese urban children was used to establish the cut-off point as 15 [37].

*Screening child anxiety-related emotional disorders (SCARED)* is used to evaluated children’s anxiety related emotional symptoms and has 41 items rated at 3 levels. A higher raw score indicates more anxiety related emotional problems. The average level in Chinese urban children suggest the cut-off point as 23 [41].

*Home quarantine investigation of the pandemic (HQIP)* is self-designed and used to obtain information regarding digital media use and activity arrangement.

We collected the average hours per day spent on the following activities, including watching TV, movies and videos, including short video (e.g. tik tok); playing video games including both computer and mobile games; and using social media software (e.g. wechat, QQ). We also collected the average number of days per week that included physical exercise, artistic and music activities (e.g. painting, singing, musical instrument playing, dancing), science and culture activities (e.g. reading, science experiment, study); and other hobbies and interests.

### Data analysis

Statistical analyses of the data were performed using SPSS Version 19.0. The demographic differences between the two groups were assessed using paired sample *t*-test for continuous data and Chi-square test for categorical data. The differences in surveys for clinical characteristics between ADHD groups with and without PDMU were assessed by using multivariate analysis of covariance (MANCOVA), with age as a covariate.

## Results

The demographic and clinical characteristics of the ADHD with and without PDMU group are presented in Table 1. There were no significant differences of gender (*χ*^*2*^ = 0.35, *p* = 0.56), ADHD subtype (*χ*^*2*^ = 1.03, *p* = 0.65), medication treatment (*χ*^*2*^ = 1.18, *p* = 0.28), and average living area (*Z* = -0.39, *p* = 0.69). While the age of the ADHD group with PDMU was significantly older (*t* = − 2.46, *p* = 0.02), and more children with comorbidity of ODD (*χ*^*2*^ = 7.36, *p* = 0.01) and Tics (*χ*^*2*^ = 4.52, *p* = 0.03) than the ADHD group without PDMU.

**Table 1:** Demographic and clinical characteristics of ADHD children with and without PDMU

Characteristics	ADHD with PDMU (*n* = 82)	ADHD without PDMU (*n* = 110)	Statistical values	*p*
Age (months) mean ± SD	136.73 ± 27.69	127.85 ± 22.21	*t* = − 2.46	0.02
Average living area (square meters) mean ± SD	32.92 ± 43.15	32.50 ± 32.24	*Z* = -0.39	0.69
Gender, n (%)				
Female	24 (29.27)	28 (25.45)	*χ*^*2*^ = 0.35	0.56
Male	58 (70.73)	82 (74.55)		
ADHD subtypes, n (%)				
Inattentive	66 (80.49)	82 (74.54)	*χ*^*2*^ = 1.03	0.65
Hyperactive-impulsive	1 (1.22)	2 (1.82)		
Combined	15 (18.29)	26 (23.64)		
Treatment, n (%)				
With medication	36 (43.90)	57 (51.82)	*χ*^*2*^ = 1.18	0.28
Comorbidity, n (%)				
ODD	42 (51.22)	35 (31.82)	*χ*^*2*^ = 7.36	0.01
Tics	31 (37.80)	26 (23.64)	*χ*^*2*^ = 4.52	0.03

*ADHD* Attention deficit hyperactivity disorder, *PDMU* Problematic digital media use, *SD* Standard deviation, *ODD* Oppositional-defiant disorder

The results of ADHD symptoms, psychosocial behaviors and academic performances of ADHD children with and without PDMU are presented in Table 2. The ADHD group with PDMU had significantly worse symptoms for inattention [F (1,189) =4.15, *p* = 0.04, *η2 p* = 0.02], oppositional defiant [F (1,189) =6.85, *p* = 0.01, *η2 p* = 0.04], conduct problem [F (1,189) =7.38, *p* = 0.01, *η2 p* = 0.04], and emotional problem [F (1,189) =14.51, *p* < 0.001, *η2 p* = 0.07], when compared with the ADHD group without PDMU. With regard to EF evaluated by BRIEF, the PDMU children presented significantly more impaired EF on shift [F (1,189) =7.01, *p* = 0.01, *η2 p* = 0.04], emotional control [F (1,189) =6.77, *p* = 0.01, *η2 p* = 0.04], initiation [F (1,189) =7.31, *p* = 0.01, *η2 p* = 0.04], working memory [F (1,189) =7.26, *p* = 0.01, *η2 p* = 0.04], plan [F (1,189) =17.09, *p* < 0.001, *η2 p* = 0.08], and behavior regulation index [F (1,189) =6.67, *p* = 0.01, *η2 p* = 0.03], metacognition [F (1,189) =10.36, *p* < 0.01, *η2 p* = 0.05], and GEC [F (1,189) =10.16, *p* < 0.01, *η2 p* = 0.05], than the group without PDMU.

**Table 2:** ADHD symptoms, psychosocial behaviors and academic performances of ADHD children with and without PDMU

Subscales	ADHD with PDMU (n = 82) mean ± SD	ADHD without PDMU (n = 110) mean ± SD	Statistical values F (1, 189)	*p*	partial η^2^
SNAP Scores					
Inattentive	17.33 ± 5.11	16.06 ± 5.38	4.15	0.04	0.02
Hyperactive-impulsive	9.49 ± 5.85	8.90 ± 5.33	3.42	0.07	0.02
ODD symptoms	10.54 ± 4.81	8.91 ± 4.63	6.85	0.01	0.04
CD symptoms	2.48 ± 2.21	1.63 ± 2.23	7.38	0.01	0.04
Emotional problem	6.28 ± 4.72	3.90 ± 3.33	14.51	< 0.001	0.07
BRIEF Factor					
Inhibition	16.57 ± 4.05	16.08 ± 3.95	1.94	0.17	0.01
Shift	13.12 ± 2.91	11.96 ± 2.67	7.01	0.01	0.04
Emotional control	17.35 ± 4.46	15.71 ± 4.08	6.77	0.01	0.04
Initiation	15.52 ± 3.01	14.22 ± 3.09	7.31	0.01	0.04
Working memory	20.93 ± 3.78	19.59 ± 3.97	7.26	0.01	0.04
Plan	27.05 ± 4.34	24.41 ± 4.43	17.09	< 0.001	0.08
Organize	13.23 ± 3.07	12.37 ± 2.96	3.44	0.07	0.02
Monitor	18.01 ± 3.08	17.46 ± 3.24	2.55	0.11	0.01
BRIEF Index					
Behavior regulation	47.05 ± 9.40	43.75 ± 9.12	6.67	0.01	0.03
Metacognition	94.74 ± 14.30	88.05 ± 15.03	10.36	< 0.01	0.05
BRIEF GEC	141.79 ± 21.86	131.81 ± 22.72	10.16	< 0.01	0.05
FES-CV					
Cohesion	6.33 ± 2.57	7.26 ± 2.00	8.05	0.01	0.04
Expression	4.95 ± 1.81	5.38 ± 1.73	3.02	0.08	0.02
Conflict	4.21 ± 2.46	3.60 ± 2.01	3.30	0.07	0.02
Independent	5.51 ± 1.50	5.41 ± 1.39	0.19	0.66	< 0.01
Achievement	5.63 ± 1.86	5.65 ± 1.73	0.01	0.93	< 0.001
Intellectual-Cultural	4.17 ± 2.11	4.62 ± 2.09	2.29	0.13	0.01
Active-Recreational	4.28 ± 2.62	4.90 ± 2.47	1.69	0.20	0.01
Morality	4.66 ± 1.64	4.63 ± 1.59	0.08	0.77	< 0.001
Organization	5.00 ± 1.46	4.89 ± 1.50	0.44	0.51	< 0.01
Control	3.54 ± 1.87	3.97 ± 2.10	3.28	0.07	0.02
Total score	48.28 ± 7.87	50.31 ± 8.85	2.95	0.09	0.02
ASLEC					
Relationship pressure	8.90 ± 4.65	5.44 ± 3.07	36.22	< 0.001	0.16
Learning pressure	9.09 ± 4.84	6.05 ± 4.00	23.16	< 0.001	0.11
Being punished	9.30 ± 5.60	5.87 ± 4.99	23.66	< 0.001	0.11
Loss	2.28 ± 3.00	1.78 ± 2.35	1.91	0.17	0.01
Adaption	2.79 ± 1.91	2.10 ± 1.75	8.65	< 0.01	0.04
Total score	36.21 ± 17.68	22.57 ± 13.65	37.38	< 0.001	0.16
SLMS					
Initiative	2.49 ± 1.60	1.48 ± 1.60	20.35	< 0.001	0.10
Awareness	2.57 ± 1.49	1.96 ± 1.41	6.36	0.01	0.03
Interesting	1.77 ± 1.24	1.46 ± 1.12	3.63	0.06	0.02
Goal	2.23 ± 1.33	1.48 ± 1.37	13.22	< 0.001	0.07
Total score	9.06 ± 3.31	6.39 ± 3.81	24.74	< 0.001	0.12
DSRSC	16.23 ± 6.11	11.45 ± 4.49	33.73	< 0.001	0.15
SCARED	25.96 ± 19.33	12.30 ± 10.54	36.02	< 0.001	0.16
Daily digital media usage					
Hours on TV/video	3.41 ± 3.02	2.87 ± 2.57	1.18	0.28	0.01
Hours on video game	2.15 ± 2.61	1.20 ± 1.74	7.14	0.01	0.04
Hours on social software	1.86 ± 2.90	0.82 ± 1.46	6.76	0.01	0.04
Days per week for doing					
Physical exercise	2.02 ± 1.95	2.77 ± 2.13	4.58	0.03	0.02
Artistic and music	0.82 ± 1.73	1.42 ± 1.90	3.60	0.06	0.02
Science and culture	1.87 ± 2.35	2.30 ± 2.48	0.25	0.62	< 0.01
Other hobbies	1.00 ± 1.85	1.63 ± 2.29	2.67	0.10	0.01

*ADHD* Attention deficit hyperactivity disorder, *PDMU* Problematic digital media use, *SD* Standard deviation, *SNAP* Swanson, Nolan, and Pelham Rating Scale, *ODD* Oppositional-defiant disorder, *CD* Conduct disorder, *BRIEF* Behavior rating scale of executive function, *GEC* Global executive composite, *ASLEC* Adolescent self-rating life events checklist, *FES-CV* Chinese version of family environment scale, *SLMS* Students learning motivation scale, *DSRSC* Depression self-rating scale for children, *SCARED* Screening child anxiety-related emotional disorders

The results of the family environment did not show significant differences except for cohesion [F (1,189) =8.05, *p* = 0.01, *η2 p* = 0.04]. The ADHD children with PDMU showed significantly more disturbances of life events for relationship pressure [F (1,189) =36.22, *p* < 0.001, *η2 p* = 0.16], learning pressure [F (1,189) =23.16, *p* < 0.001, *η2 p* = 0.11], being punished [F (1,189) =23.66, *p* < 0.001, *η2 p* = 0.11], adaption [F (1,189) =9.65, *p* < 0.01, *η2 p* = 0.04], and total situation [F (1,189) =37.38, *p* < 0.001, *η2 p* = 0.16] compared to the ADHD children without PDMU. Consequently, the ADHD children with PDMU had significant more problems on learning motivation [F (1,189) =24.74, *p* < 0.001, *η2 p* = 0.12], including initiative [F (1,189) =20.35, *p* < 0.001, *η2 p* = 0.10], awareness [F (1,189) =6.36, *p* = 0.01, *η2 p* = 0.03], and goal [F (1,189) =13.22, *p* < 0.001, *η2 p* = 0.07], compared to ADHD children without PDMU.

The total score on DSRSC [F (1,189) =33.73, *p* < 0.001, *η2 p* = 0.15] and SCARED [F (1,189) =36.02, *p* < 0.001, *η2 p* = 0.16] was significantly worse in ADHD with PDMU group, compared with ADHD without PDMU group. In the ADHD group with PDMU, children had a significantly higher positive relationship in DSRSC (44/82 vs. 30/110, *χ*^*2*^ = 33.55, *p* < 0.001) and SCARED (43/82 vs. 15/110, *χ*^*2*^ = 13.81, *p* < 0.001) than children in the ADHD group without PDMU. However, with regard to the diagnosis with depression disorder and generalized anxiety disorder (GAD), there were 2 children with depression disorder in ADHD with PDMU group, none in ADHD without PDMU group. Neither group had ADHD children met GAD diagnosis criteria. The diagnose differences with depression and GAD between were not significant between two groups.

Our investigation found that there were significant differences on media usage patterns and daily life arrangement between two groups. The ADHD children in PDMU group spend significantly more time on digital media use regardless of whether it was video game [F (1,189) =7.14, *p* = 0.01, *η2 p* = 0.04] or social media software [F (1,189) =6.76, *p* = 0.01, *η2 p* = 0.04]. On the other hand, the ADHD children in PDMU group spend significant less days on physical exercise [F (1,189) =4.58, *p* = 0.03, *η2 p* = 0.02], however, there were not significant differences on the days spend on artistic and music [F (1,189) =3.60, *p* = 0.06, *η2 p* = 0.02], science and culture [F (1,189) =0.25, *p* = 0.62, *η2 p* < 0.01], and other types of hobbies [F (1,189) =2.67, *p* = 0.10, *η2 p* = 0.01].

## Discussion

The study found that the ADHD children with PDMU suffered more severe core symptoms, negative emotions, EF deficits, damage of the family environment, pressure from life events and lower motivation to learn.

### ADHD core symptoms

ADHD children with PDMU showed significantly more core symptoms of inattention than ADHD children without PDMU. Previous studies suggested that there was a link between PDMU and attention deficits [8, 43]. On one hand, children with severe attention deficit might be more likely to be passively attracted to digital media products as distracting and smoothing themselves to compensate for diminished social abilities or academic difficulties [29]. On the other hand, too much digital media use time could also interfere with the ability to concentrate. Recent research raised the concern that interactivity and reflective reactivity on mobile media might contribute to the development of ADHD symptoms [32]. Screen time also might hinder the availability for activities that were considered better for stimulating cognitive abilities and a longer attention span [27].

### Emotional problems

ADHD children with PDMU, as compared with ADHD children without PDMU, showed an increase of emotional problems including both higher scores and abnormal rates when using the SCARED and DSRSC methods of measurement. Many previous studies, although inconclusive, had revealed that children who spend more time online were more likely to be depressed [20]. The diagnosis of both anxiety disorder and depression disorder, however, did not show a significant difference in our study. This might suggest that ADHD children with PDMU were more likely to experience negative emotions due to inappropriate use of digital media products and not due to the effects caused by psychiatric disease as anxiety or depression. It was plausible that social media lead youths who feel lonely to compensate by engaging in passive internet use, such as scrolling other people’s accounts, however this might end up increasing their depressed mood [6]. Gaming and social media seemed to offer effective methods of digital communication for anxious children, however sharing emotions and experiences through face-to-face interaction with peers was lost [19], and might lead to a struggle with real-life social interactions resulting in an anxious social disposition.

### Executive function

EF deficits have been shown to closely related to ADHD in many studies [34, 36, 48], and has been shown to result in extensive and severe functional impairments [38]. This study found EF was much worse in the ADHD with PDMU group as compared to the ADHD without PDMU group in the areas of both elements and total situation. Exposure to observational media such as television might have a negative impact on child’s EF performance [21]. Additionally, some researchers found that both television exposure time and content were related to children’s current EF along with their EF performance later in life [26]. Media exposure therefore might damage EF abilities, and as viewed from another angle, poor EF might cause children to likely lose control of digital media use. EF deficits associated with ADHD such as lack of self-control, self-regulation, and behavioral inhibition would lead to difficulties in daily life management [24], including regulation of media and internet use [25].

### Parent-child relationship

While their family environment exhibited worsened cohesion, the ADHD children with PDMU showed severe symptoms of ODD, as compared with the ADHD children without PDMU. This indicates that there were more problems such as confrontation and disobedience, within the parent-child relationship. One study that included Chinese families found that parent-child relationship helped to mediate adolescent problematic mobile phone use and parental phubbing [28]. The healthy function of a family, needing balanced cohesion and flexibility, required both parents and children to maintain low levels of digital device usage. In contrast, family function would be damaged when parents showed high levels of media usage and also allowed children high usage too [7]. In fact, both the internet and mobile phone could interact with relationship and individual’s function of every family member [35].

### Learning motivation

The ADHD children with PDMU exhibited lower learning motivation, higher stress on life, interpersonal, and learning problems as compared with the ADHD children without PDMU. In fact, one study that included Chinese adolescents found that the participants with problematic use of mobile phone presented more stress from life events and less motivation toward school work as compared with the healthy controls [12]. This negative association between screen-based activities and academic performance, which further affected work opportunity, might be due to the idea that screen media use played a key role in cognition, including the brain processes involved in knowledge, intellect and action which ultimately affected academic abilities and achievements [15]. Because of this, screen-based activities should be supervised and reduced to improve academic performance [1].

### Physical activities

The ADHD children with PDMU spend considerably more screen time on both video games and social media software, and less time on physical exercising, than the ADHD children without PDMU. Studies have revealed that both video games [25] and social media use [10] had negative consequences on attention and regulation. Furthermore, previous studies have investigated the different types of screen-based activities for their individual impact on children’s function. They found that television viewing and video game playing appeared to be the activities most negatively associated with academic outcomes particularly [1]. This strongly suggests that parents should manage digital media exposure, but should also promote increased physical activity in children. One study has reported that less screen time and more frequent vigorous physical activity was associated with a lower risk of depression, anxiety, low self-esteem, and life dissatisfaction, which suggested that reducing screen time while increasing physical activity might lead to good mental health outcomes [16].

It is important to manage children’s use of digital media products, making prevention and early intervention, because screen use is now so ubiquitous. Greater access to media and reduced parental supervision have contributed to prolonged leisure screen time in Chinese children with adolescents [46]. To prevent disruptions to a child’s daily family, social interaction, school performance, and physical functioning, it is necessary to pay attention to the signs of PDMU and seek early treatment [30].

### Limitation

This study was carried out during the special period of COVID-19 epidemic and due to the home quarantine policy, along with the fact that participants were families who seek medical help voluntarily, confounding factors such as comorbidities and medication use were not controlled. This special period could be seen as an extended vacation because the long break from the classroom, however, the influence of COVID-19 could still affect children’s emotion and stress. This study may reveal the relationship between PDMU and ADHD children’s symptoms, emotional and behavioral problems, parent-child relationship, and family environment, however, it is difficult to explain any causal relationship. The effects of PDMU and problematic behaviors, negative emotions, and dysfunctional family relationship might be interacted with each other. Due to the special period, our study only included ADHD children, but not healthy control children, which could not better explain the relationship between ADHD symptoms and internet use in the whole children population. Finally, although this study suggested a variety of negative influences of PDMU, feasible and effective intervention need to be further explored in future study.

## Conclusion

This study, taken as a whole, not only reveals the connection between the use of digital media and ADHD symptoms, emotions, EF, learning motivation and family environments, but also shows enhanced problems in ADHD children with increased exposure to digital media during the epidemic quarantine period. ADHD symptoms, behavioral problems, EF impairments, and family environment problems all showed increases with increased use of digital media. Although no causal relationship is shown by our study, parents should consider taking action to limit digital media exposure to prevent family, emotional, and learning disruptions. For example, during break from the classroom, parents of children with ADHD should adhered to prescribed medication for ADHD symptom management, while attempting to provide a good family environment, finding ways to manage negative emotions, increase physical exercise, and reduce the use of digital media products.

## Data Availability

The author could provide raw data if needed.
